# Propolis as an Adjuvant in the Healing of Human Diabetic Foot Wounds Receiving Care in the Diagnostic and Treatment Centre from the Regional Hospital of Talca

**DOI:** 10.1155/2019/2507578

**Published:** 2019-09-12

**Authors:** Verónica Mujica, Roxana Orrego, Roberto Fuentealba, Elba Leiva, Jessica Zúñiga-Hernández

**Affiliations:** ^1^Escuela de Medicina, Facultad de Medicina, Universidad Católica del Maule, Talca, Chile; ^2^Departamento de Bioquímica Clínica e Inmunohematología, Facultad Ciencias de la Salud, Universidad de Talca, Talca, Chile; ^3^Programa de Doctorado en Investigación y Desarrollo de Productos Bioactivos, Universidad de Talca, Chile; ^4^Laboratorio Clínico Loncomilla LTDA, Talca, Chile; ^5^Farmacología, Escuela de Medicina, Universidad de Talca, Talca, Chile

## Abstract

**Objective:**

Diabetic foot wounds are a relevant diabetes complication and a major health problem. It has been described that propolis has health benefits due to its anti-inflammatory, antioxidant, and support in the healing process. The current study assessed the effect of propolis as an adjuvant in the healing of human diabetic foot ulcers. This was evaluated in a randomized placebo-controlled study of subjects receiving care in the Diagnostic and Treatment Centre from the Regional Hospital of Talca, Chile.

**Research Design and Methods:**

Randomized subjects received ambulatory healing treatment for diabetes foot wounds with propolis spray (3%), which was applied to cover the entire wound surface each time it was dressed from week 0 until cicatrization or 8 weeks as a maximum. Two serum samples were taken (day 0 and end of the study) for cytokine and oxidative stress analyses. Also, macro- and microscopy were analyzed in the process of wound healing.

**Results:**

The study comprised 31 subjects with type 2 diabetes in treatment for diabetic foot wounds in the Diagnostic and Treatment Centre from the Regional Hospital of Talca. Propolis promotes a reduction of the wound's area by an average of 4 cm^2^, related to an increase in the connective tissue deposit compared to the control. Also, propolis increased the glutathione (GSH) and GSH/glutathione disulfide (GSSG) ratio (*p* < 0.02), depleted tumor necrosis factor- (TNF-) *α*, and increased interleukin- (IL-) 10 levels. Topical propolis did not modify the biochemical parameters in the serum of the studied subjects.

**Conclusions:**

The topical use of propolis turned out to be an interesting therapeutic strategy as an adjuvant in the care of diabetes foot wounds due to its ability to improve and promote healing based on its anti-inflammatory and antioxidant profile. This trial is registered with NCT03649243.

## 1. Introduction

Diabetes Mellitus is a chronic disease with high prevalence in the world; it is estimated that on an average, 7% of the world population are diabetics [1]. Diabetes presents global mortality rates of 9%, which equals to 4 million deaths per year [2]. Patients with diabetes have higher rates of premature death, functional disability, and coexistence with other diseases compared to healthy subjects. The progressive increase of this pathology has been associated with a rise of diabetic's chronic complications such as foot amputations [3, 4]. Overall, diabetic foot is the first cause of nontraumatic amputation [5] and affects about 15% of all patients with Diabetes Mellitus, even though most cases are preventable [6].

Diabetic foot ulcers are not only a patient problem but also a major healthcare concern throughout the world and are one of the common and serious complications in diabetic patients. The treatment of complications in diabetic ulcers is difficult and expensive. Patients usually need to take long-term medications or become hospitalized for an extended period of time [7]. These diabetes complications are associated with cardiovascular risk factors such as high blood pressure, dyslipidemia, and obesity, which can contribute to arterial obstruction. This together with orthopedic deformation is one of the most important pathological conditions that lead to development of diabetic foot [8, 9]. Nevertheless, the hyperglycemia status is the most relevant factor in the development and worsening of diabetic foot pathology, producing multiple metabolic and molecular changes such as sorbitol gain with increased glycation-end products and oxidative damage and increased in kinase C protein activity. These factors are directly related to diabetic microangiopathy [10]. All of these processes could be present in the eyes, kidneys, nervous system, and others. Specifically, the skin presents most of these alterations with changes in temperature, hydration, and dermis perfusion. These symptoms are caused by the neuropathy (autonomic and sensitive) that seriously affects the extremities of these patients. As a whole, both macro- and microangiopathy are responsible for the difficulty in healing wounds and favor infections in the diabetic foot. The presence of aggressions and/or trauma in diabetic foot, even slightly, can lead to development of ulcerations that in a high percentage proceed to amputations [11].

Stimulating ulcer cicatrization represents a permanent challenge for health services around the world. This healing is determined by multiple factors that involve molecular reactions influenced by the microenvironment of the wound, the persistent inflammation, ischemia, oxidative stress, and infections [12]. There are multiple factors that impair the recovery of a diabetic wound, including vascular insufficiency, deregulation of inflammatory processes, and angiogenic responses, among others. Persistence of inflammation and neutrophil infiltration is characterized by the chronic upregulation of proinflammatory cytokines and superoxide anions in the diabetic wounds [13]; all of these may interfere with the normal process of wound recovery.

There are multiple natural products with potential benefits in the healing process such as propolis (a resin produced by bees), which has been attributed to beneficial effects on human health, specifically for its antioxidant, antimicrobial, and immunomodulatory capacity [14, 15]. In diabetes clinical studies, there is a lack of evidence that shows the specific effects and mechanisms of these natural products. Previous published studies of this research group demonstrated the beneficial effect of propolis on oxidative stress in subjects treated for three months with oral propolis [16]. Considering this, the objective of the present study was to evaluate the effect of propolis as an adjuvant on the healing of human diabetic foot wounds receiving care in the Diagnostic and Treatment Centre from the Regional Hospital of Talca, Chile.

## 2. Research Design and Methods

### 2.1. Participants

All of the diabetic patients with foot wounds receiving ambulatory treatment from the Diagnostic and Treatment Centre at the Regional Hospital of Talca in October 2015 to March 2016 who met the inclusion criteria were invited to participate in this study. The ethics committee of the Maule Health Service approved this project on September 11^th^ of 2015 (Folio number 2015-c03), and it was also approved by the Bioethical Committee of Universidad de Talca (Folio number 2015-095-EL). All included patients signed an informed consent. The inclusion criteria were type 1 or 2 diabetes with complicated foot diabetic wounds under complete treatment in the diabetes program and between 18 and 80 years of age (only type 2 diabetes subjects accepted to be part of the study). The exclusion criteria were (i) propolis allergy, (ii) critical ischemia, (iii) uncontrolled severe infection, and (iv) psychosocial conditions that impede regular attendance for health assistance. A total of 31 subjects were eligible for this study and follow-up for a maximum of 8 weeks. Twenty voluntary subjects were allocated in the propolis group, and 11 voluntary subjects were allocated in the control group. At the end, three patients discontinued the study (flow chart of enrolment in supplementary [Supplementary-material supplementary-material-1]).

### 2.2. Propolis

The propolis (Beepolis®) used was 3% in propylene glycol preparation manufactured by a bee product company in the Maule Region of Chile (Health Authorization no. 639-18/08/2009, Laboratories Rotterdam, Maule, Chile). Propolis spray was applied to cover the wound surface in each dressing from week 0 until cicatrization or 8 weeks as a maximum, whichever occurred first.

### 2.3. Wound Evaluation

Macroscopic aspect (*wound area measurement*): the nurse who dressed the wound and applied the propolis was the same for all subjects and did not participate in the result analysis. Control subjects received the same nursing care without the addition of propolis spray (diabetic foot wound care medications are summarized in [Supplementary-material supplementary-material-1]). The nurse evaluated the wound by taking a photograph and measured the area (large × width = cm^2^) with acetate tracing at the beginning of the treatment (week 0) and at the end of the study. Microscopic aspect (*histopathology evaluation*): representative fragments of wound sections (biopsy) were fixed in 10% buffered formalin and embedded in paraffin. Formalin-fixed paraffin-embedded tissues were stained with Masson's trichrome according to the fabricant's instructions (Merck, Germany). The collagen fibers were marked with light blue, while the cellular component was marked with red stain. Fibrous tissue areas were quantified using an arbitrary scale (ACT) based on the Ishak fibrosis score [17] (see Supplemental [Supplementary-material supplementary-material-1]).

### 2.4. Serum and Tissue Evaluation

#### 2.4.1. Biochemical Parameters in Serum

Glycaemia was measured using a colorimetric enzymatic hexokinase test (Glucose-Custom Biotech), insulin by electrochemiluminescence immunoassay (insulin ECLIA), HbA1c (glycosylated hemoglobin A1c) by a turbidimetric inhibition immunoassay (Tina-quant hemoglobin A1c Gen.2®), and C-reactive protein (CRP) by highly sensitive turbidimetric immunoassay (cardiac C-reactive protein (Latex) High Sensitive®). The analyses were measured in a Cobas c311 autoanalyzer (Roche, Switzerland).

#### 2.4.2. Oxidative Status

*(1) TBARS Measurement*. Thiobarbituric acid reactive substances (TBARS) were measured according to Knodell et al. [18]. Briefly, 250 *μ*L of each serum was incubated with 0.67% thiobarbituric acid and 50% trichloroacetic acid for 30 min at 90°C and centrifuged at 2500 rpm for 15 min. The supernatant was used to measure TBARS at 530 nm in a Multiskan Go microplate reader (Thermo Fisher Scientific, USA). The results were expressed in nmol/mL using malondialdehyde (MDA) as a standard curve (Sigma-Aldrich, USA).

*(2) Glutathione (GSH) Measurement*. The GSH level was measured using metaphosphoric acid for protein precipitation and 5,5-dithiobis 2-nitrobenzoic acid (DTNB) (Sigma-Aldrich) for color development at 412 nm. 40 *μ*L of whole blood from EDTA tube was mixed with 760 *μ*L of distilled water. 1200 *μ*L of precipitating solution (1.67 g of glacial metaphosphoric acid, 0.2 g of EDTA, and 30 g of NaCl in 100 mL of distilled water) was added to this mixture and centrifuged at 3500 rpm for 10 minutes. To 250 *μ*L of the supernatant, 1000 *μ*L of phosphate buffer and 125 *μ*L of DTNB were added. This solution was used to measure GSH spectrophotometrically (Multiskan Go, Thermo Fisher Scientific). The values were expressed as mg/dL for serum analysis and *μ*mol/gr for tissue analysis.

#### 2.4.3. Inflammatory Status

*(1) Serum Cytokine*. Serum tumor necrosis factor- (TNF-) *α* and interleukin- (IL-) 10 were measured by an ELISA technique (Thermo Scientific) according to the manufacturer's instructions and were determined spectrophotometrically (Multiskan Go, Thermo Scientific) at 450 nm, and the concentration was calculated against a standard curve; the levels were expressed as pg/mL.

*(2) Tissue Cytokine*. TNF-*α* and IL-10 were analyzed by quantitative real-time PCR (RT-qPCR). Before PCR, total RNA of each sample was processed with a RNase-free DNAse kit (Ambion, Life Technologies, USA), NanoDrop (Thermo Scientific) RNA was reversed by Revert Aid Reverse Transcriptase (Thermo Scientific), and RT-qPCR was performed for the following genes: TNF-*α* 5′-GGTTCCGTCCCTCTCATACA-3′ forward and 5′-AGACACCGCCTGGAGTTCT-3′ reverse primer and IL-10 5′-TGGAGTGAAGACCAGCAAAG-3′ forward and 5′-GGCAACCCAAGTAACCCTTA-3′ reverse primer, with GAPDH as a housekeeping gene 5′-TTGTGAAGCTCATTTCCTGGTA-3′ forward and 5′-GGCCTCTCTCTTGCTCTCAGTA-3′ reverse primer. The assay was performed in a thermo cycler (Stratagene Mx3000P, Agilent Technologies, USA). The thermal cycle conditions were 95°C for 5 minutes, 40 cycles of 95°C for 15 seconds, 60°C for 45 seconds, and finally, a dissociation cycle. Efficiency of every primer set was calculated through a serial dilution of a cDNA sample from 10^−1^ to 10^−8^. The gene expression level was measured on a standard curve; additionally, relative change was calculated using 2^-*ΔΔ*Ct^ methods and normalized to GAPDH.

### 2.5. Statistical Analysis

All values correspond to mean ± SEM or standard deviation (SD). The data were evaluated with GraphPad Prism 6® software (La Jolla, USA). The statistical analysis included intragroup *t-*student analysis and one-way ANOVA followed by the Mann-Whitney test for unpaired data. A *p* value of <0.05 was considered statistically significant.

## 3. Results

### 3.1. Description of General Characteristic Demographic

A total of 31 patients were eligible for this study and provided informed consent.  Table 1 summarizes the demographic characteristics of the study population and the treated ulcer details. The control group consisted of five men and three women with an average age of 58.8 ± 6.34 years and a diabetes diagnosis of 7.6 ± 3.5 years ago. The propolis group consisted of 16 men and 4 women with an average age of 60 ± 11.2 years and a diabetes diagnosis of 11.8 ± 6.4 years ago. These parameters did not show significant differences between groups. All the subjects were screened for the presence of other pathologies and concomitant therapies (see [Supplementary-material supplementary-material-1]); pharmacological treatment remained constant throughout the entire study and the control of all their pathologies with the appropriated specialist physician. Hematological and serum parameters were measured for the groups' pre- and posttreatment (Table 2); postprandial glycaemia was measured at the beginning and at the end of the study; the average for the control was 320 mg/dL (range of 157 to 716 mg/dL) at time 0 and 196 mg/dL (range of 87 to 349 mg/dL) at the time of wound healing with nonstatistical significance (*p* = 0.0794). For propolis-treated subjects, the average at time 0 was 213 mg/dL (range of 65 to 384 mg/dL) and 215 mg/dL (range of 71 to 598 mg/dL) at the end of the study with nonstatistical significance among the data. The mean value of HbA1c (glycosylated hemoglobin A1c) was 10.3 and 9.1% for the control group but 9.8 and 9.3% for the propolis-treated patients at the beginning and at the end of the study, respectively, with nonstatistical significance among the groups. Other parameters measured were creatinine, total cholesterol, triglycerides, hematocrit, hemoglobin, and white blood cells, with no significant differences in time. Also, the levels of high sensitive C-reactive protein (usPCR) were analyzed; no differences were found among the groups ([Supplementary-material supplementary-material-1]).

### 3.2. Wound Analysis

**Figure 1:**
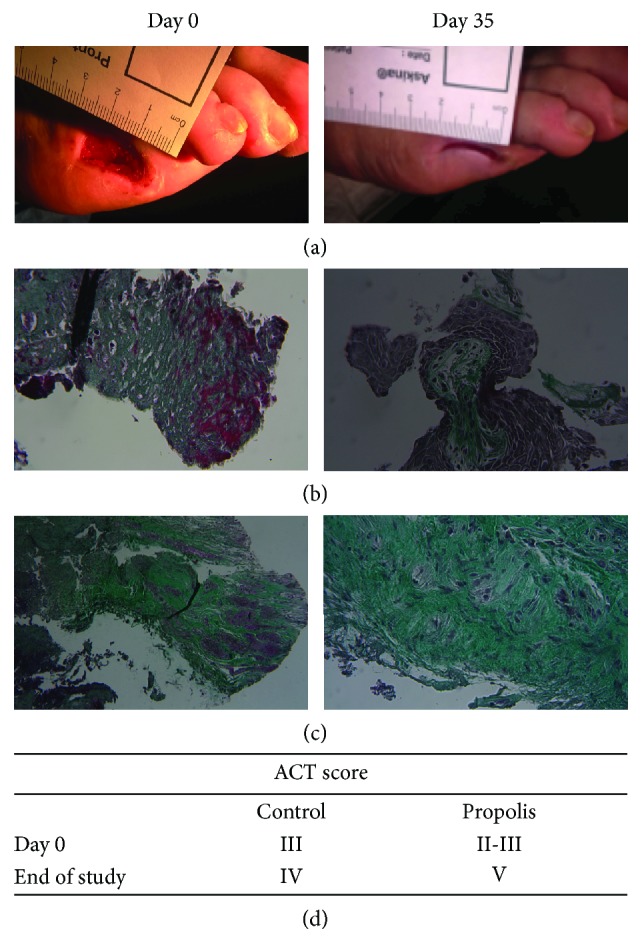
Representative images of a representative photograph of wound healing from a subject treated with propolis (a), control (b), and propolis (c) of foot wound biopsies stained with Masson's trichrome. (c) Day 35 means the last tissue biopsy sample for that patient, and (d) is the average of the ACT score determinates for all the samples.

*Macroscopic aspects*: the data shows a significant difference (*p* = 0.0317) in the wound healing of the propolis group in relation to the control group; specifically, there was a decrease in the wound area by an average of 4 cm^2^ in the propolis group compared with the control group, which reduced 3 cm^2^ (Figure 1(a)). *Microscopic analysis* (*histopathological*): to evaluate whether the propolis treatment had an effect on collagen deposition and formation of fibrotic tissue (potential scar), histological staining with Masson's trichrome was performed, as observed in Figures 1(b) and 1(c). At the beginning, wound tissues showed the presence of 70 to 80% of connective tissue in the biopsies from both groups. At the end of the study, the presence of fibrosis and connective deposit was increased to 95% in propolis groups compared to an average of 80-85% in controls. When the ACT scale was applied (Figure 1(d)), it was possible to observe that the control group changed score III to IV and propolis from II-III to V. Together with the biopsy, the presence of microorganisms in the wound was analyzed (see Supplemental [Supplementary-material supplementary-material-1]); the most prevalent bacteria found was *S. aureus* with almost 30% in both groups, and none of the subjects had infections derived from fungi.

#### 3.2.1. Oxidative Status

**Figure 2:**
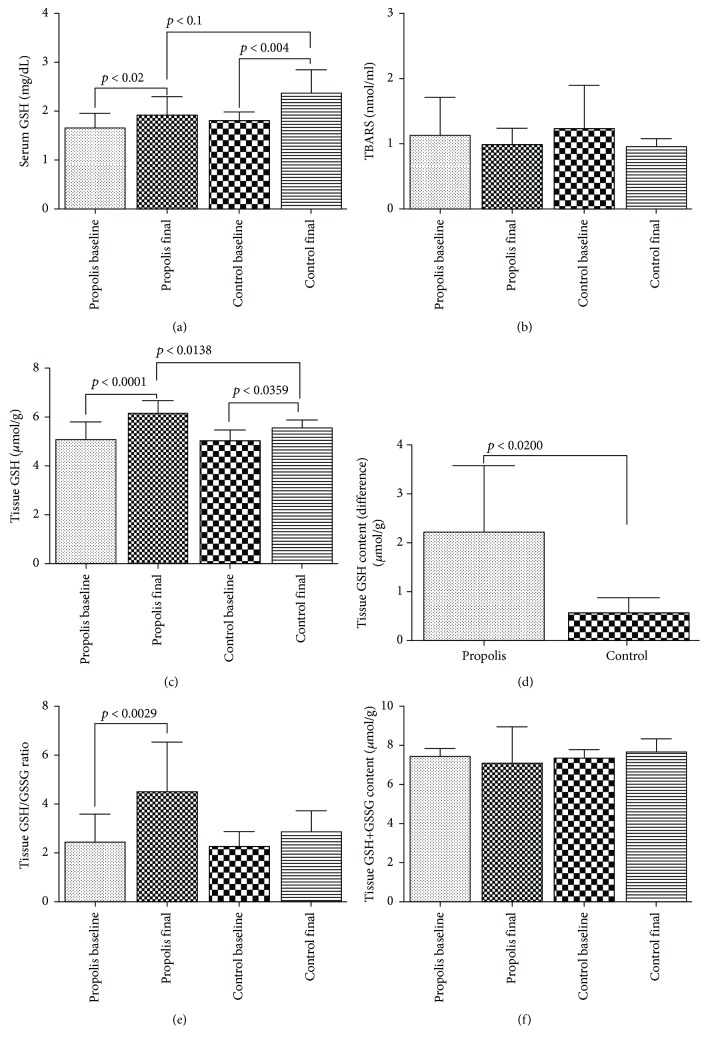
Oxidative status. Serum analysis of GSH (a) and TBARS (b). Tissue analysis of GSH (c), net change of GSH (d), GSH/GSSG (e), and total tissue content of GSH+GSSG (f). Results are expressed as mean ± SD for 8 control subjects, and 20 propolis subjects (*t*-student and Tukey's posttest). *p* < 0.05 was considered significant.

The serum oxidative stress analysis shows that GSH increased in the time in both groups (*p* < 0.02 and *p* < 0.04, respectively) (Figure 2(a)). The control group displayed a higher increase of GSH than the propolis group at the end of the study (*p* < 0.01). Serum TBARS showed nonsignificant differences between the control and propolis groups (*p* < 0.66) (see Figure 2(b)), and the differences showed no significant statistical changes. GSH and GSH/glutathione disulfide GSSG were determined in tissue (see Figures 2(c)–2(e)); GSH increased in time in the control and propolis groups (*p* < 0.03 and *p* < 0.0001, respectively), and GSH increased more in the propolis group than the control at the end of the study in all subjects (*p* < 0.01). Also, GSH tissue content was determined for analyzing the net change of GSH (Figure 2(d)), evidencing and increasing the total content of GSH in the subjects treated with propolis (*p* < 0.05). The GSH/GSSG ratio was enhanced in time in the propolis group (*p* < 0.002) (Figure 2(e)). The control patients showed nonsignificant differences in the GSH/GSSG ratio. Additionally, it was verified that GSH or GSSG were not lost during the entire study (Figure 2(f)).

#### 3.2.2. Inflammatory Status (Cytokines Analyses): TNF-*α* and IL-10

**Figure 3:**
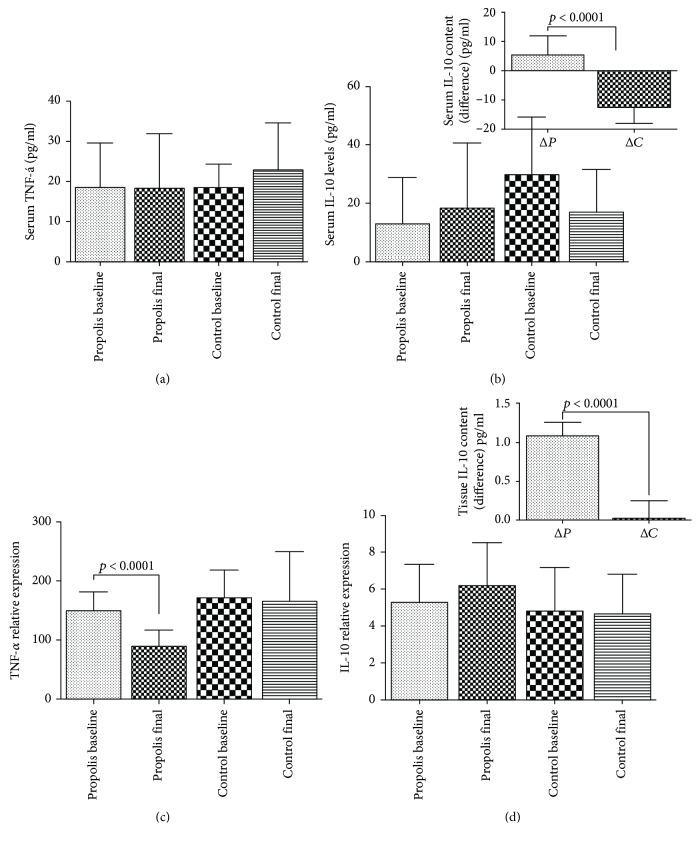
Cytokine pattern. Serum analysis of TNF-*α* (a) and IL-10 (b). Tissue analysis of TNF-*α* (c) and IL-10 (d). In the insets, it is possible to observe the net change of IL-10: Δ*P* equal propolis final minus propolis at time zero; Δ*C* equal control final minus control at time zero. Results are expressed as mean ± SD for 8 control subjects and 20 propolis subjects (*t*-student and Tukey's posttest). *p* < 0.05 was considered significant.

The inflammatory status was determined by the levels of TNF-*α* and IL-10 in serum and in the wound tissue (Figures 3(a) and 3(b), respectively). The levels of TNF-*α* and IL-10 showed no serological changes both in the control and in the propolis-treated group, but when these cytokines were extracted from the site of the injury, it was possible to observe that (i) TNF-*α* decreased in time in the propolis group (*p* < 0.0001), a situation not observed in the control group, where the TNF-*α* levels remain constant over time (*p* < 0.5197) (Figure 3(c)). (ii) IL-10 did no show significant changes over time in the control and/or propolis group (*p* < 0.9744 and *p* < 0.2281, respectively), but when the net change over time was analyzed, it was possible to observe in the treatment group a 100% increase of IL-10 (see inset in Figure 3(d)), an increase not seen in the control group.

## 4. Discussion

Chronic wounds are a rising problem in healthcare systems worldwide as the population ages and experiences increases in the incidence of obesity and diabetes. These wounds are difficult to heal, and treatment is often lengthy and expensive [19]. Multiple efforts have been made to improve the treatment. Wound research, therapies, and treatment options are a critical challenge. Nevertheless, some studies have been carried out for understanding the molecular and cellular *milieu* of chronic wounds in order to remove the main barriers that prevent their healing [20].

There is an increasing interest in the use of natural products in modern medicine as part of disease and patient management. Bee products are natural and have diverse applications in medical fields for the treatment of various diseases. The identification of bee products that may enhance skin repair can contribute to a better understanding of the wound healing process and generate a new strategy to combat chronic wounds [21]. The present study shows favorable changes in the patients that received topical treatment of propolis at the site of the wound, healing better than those not treated. Previously, it has been reported that propolis is well tolerated and reduces the area of the ulcer by an average of 41% vs. 16% in controls (applied Wagner's classification weekly) [15] and reduces the size of ulcers (four weeks) with grades 1 and 2 [22], proving for the first time that topical propolis may enhance wound closure. The evolution of patients treated with topical propolis in our research for an average of 8 weeks showed a 25% reduction in the wound area. We also analyzed the histopathological deposit of the extracellular matrix in the foot wound biopsies by Masson's trichrome stain. Masson's trichrome has been extensively used to analyze collagen remodeling and histological examination in the promotion of wound healing in models of diabetes [23]. Thus, it takes into consideration that the regeneration phase of the wound involves extensive tissue remodeling by replacing proteoglycan and collagen molecules. This results in stronger tissues where the collagen is one of the most important events in wound healing. In diabetes, collagen fibber synthesis was impaired and it was accompanied by an increased apoptosis of fibroblasts [24]. Topical propolis administration allowed a better connective tissue deposit with a favorable trend to regeneration in comparison with the nontreated group.

The persistence of hyperglycemia in diabetes is an important cause of increased production of reactive oxygen species (ROS), which enhance oxidative stress and become the main factor of cardiovascular complications in diabetes. Moreover, diabetes is characterized by the presence of inflammatory mediators such as cytokines, growth factors, and free radicals that may accelerate the development of diabetes. Thus, inflammatory and oxidative events seem to act together in the development of chronic pancreatic inflammation leading to the deterioration of its function. Several lines of evidence indicate that ROS production activates signaling pathways that promote angiogenesis [25]. Hyperglycemia is an important factor for the intense oxidative stress in diabetes, and the toxicity induced by glucose autoxidation is likely to be one of the important sources of ROS. Additionally, lipid peroxidation plays an important role in the production of free radicals and oxidative stress in diabetes [26]. Preceding authors have shown that bee honey and their variants can ameliorate the oxidative parameters in diabetic animal models. Particularly, bee honey reduced superoxide dismutase (SOD) and decreased catalase (CAT) and MDA levels [26–29]. Also, GSH levels and GSH/GSSG ratio were significantly elevated, and honey did not increase the levels of glutathione peroxidase [28]. On the other hand, it has been demonstrated that propolis administration has beneficial effects. El-Sayed et al. [30] showed that the ethanolic extract of propolis in (streptozocin) STZ-treated rats generated a marked reduction of GSH and CAT (66% and 31%, respectively) in serum and SOD (54%) in the pancreas. The same parameters were measured in kidney tissues of animals induced by diabetic nephropathy where the oral administration of propolis extract in doses of 100, 200, and 300 mg/kg improved the serum glucose, lipid profile, MDA, and renal function tests. Kidney GSH, SOD, and CAT were significantly increased while MDA was markedly reduced [31]. This would suggest a strong antioxidant effect of propolis, which can ameliorate oxidative stress and delay the occurrence of diabetic complications.

Propolis is rich in antioxidants such as polyphones and flavonoids. Antioxidant activity of propolis occurs with high amounts of phenolic compounds, and weak activity occurs in low amounts. It has also been reported that flavonoids reduce blood glucose levels [26]. The topical administration in our protocol generated a change in the tissue oxidative parameters with a significant increase in tissue GSH levels and GSH/GSSG ratio, which is related to an action of propolis over the site of the wound and not a systemic action, taking in consideration that the hyperglycemia was not modified in the serum of the subjects, but in the serum, the oxidative parameters have a trend to normalization, must probably this is due to an improvement in the wound healing observed in all the patients. According to the previously discussed, these parameters are high predictive criteria for focal oxidative stress although there are no previous studies that evaluated the diabetic foot with these markers. We can propose that the improvements in foot wounds are directly related to the local antioxidant potential previously described for propolis.

Together with the changes described for oxidative stress, we found changes in the local inflammatory parameters with significant modification in TNF-*α* levels and an increase in IL-10. It should be considered that diabetes-induced ulcers, at least in experimental models, display impaired profiles of proinflammatory/anti-inflammatory factors. This phenomenon is associated with a delay in the resolution phase of the healing process because aberrant messages are sent to T- and B-lymphocytes and macrophages, thereby impairing reepithelialization and remodeling. This is normally carried out by platelets, macrophages, epitheliocytes, and fibroblasts, representing the final phase of healing associated with physiological inflammation [32]. Natural antioxidants play various biological roles in the treatment of diabetic complications, including impaired wound healing and T-cell immune responses in offspring born to diabetic mothers, as well as the treatment of other diseases [32–34]. Previously, the group of Al Ghamdi et al. [33] showed that the ethanol-soluble derivative of propolis administered to mice with diabetes induced by STZ significantly increased the circulating lymphocyte count. This was associated with the restoration of the aberrant elevated levels of proinflammatory cytokines IL-1*β*, IL-6, and TNF-*α* and the normalization of the reduced levels of IL-2, IL-4, and IL-7, concluding that propolis impaired lymphocyte proliferation and migration towards chemokines to maintain an efficient lymphocyte immune response.

There are several lines of evidence posing the Nuclear Factor kappa B (NF*κ*B) as a key regulator in the crosstalk among the pathways leading to type 2 diabetes. It was documented that NF*κ*B is activated via phosphorylation of inhibitor NF*κ*B (I*κ*B) leading to its ubiquitination and proteasomal degradation. Such a reaction will unmask the nuclear localization signal of NF*κ*B, and once in the nucleus, it will activate several genes that regulate proliferation, apoptosis, angiogenesis, and inflammation [35]. Furthermore, obesity activates the transcription factor NF*κ*B, which increases the risk for diabetes. It has been shown that NF*κ*B pathway inhibition exerts a beneficial effect on type 2 diabetes [36]. Considering the above, it was discovered that TNF-*α* is overexpressed in the adipose tissues of obese mice, thereby establishing a clear link between obesity, type 2 diabetes, and chronic inflammation [35]. Propolis and its constituent caffeic acid showed a higher antioxidant activity and inhibited nitric oxide production in macrophages without cytotoxicity by blocking NF*κ*B and Mitogen-Activated Protein Kinase (MAPK) activation in macrophages. This did not induce hepatotoxicity at concentrations with strong anti-inflammatory potential [32]. It would be interesting to analyze the role of propolis over NF*κ*B in diabetic wound foot.

It is important to highlight that we have not found any changes in the values of hemoglobin A1c (HbA1c) nor glycaemia among the groups. Thus, this would respond to the topical administration of propolis and that all the patients in both groups maintained the normal adjustment of their pharmacological treatment. These findings support that the observed tissue levels are due to the effect of the topical propolis and are not derived from systemic interventions.

## 5. Conclusion

Propolis promotes the closure of diabetes foot wound and the reduction of the injury area related to an increase in the extracellular matrix deposit, which helps the cicatrization. Topical propolis contributes to oxidative stress equilibrium by enhancing GSH and GSH/GSSG ratio and decreasing inflammation mediated by the depletion of TNF-*α* and the enhancement of IL-10 in the injury area. Propolis seems to be an attractive adjuvant tool for the management of diabetic foot wounds that could offer a wide cost-benefit ratio.

## Figures and Tables

**Table 1:** Demographic and wound characteristics.

	Control	Propolis	*pvalue*
Subjects number	8	20	
Age (mean ± SD)	58.8 ± 6.34	60 ± 11.2	0.739
Gender			
Female	3 (37.5%)	4 (20%)	
Male	5 (62.5%)	16 (80%)	
Diabetes duration (year) (mean ± SD)			0.154
	7.6 ± 3.5	11.8 ± 6.4	
Reduction wound area (cm^2^)			
Mean	3.03	4.0	^∗^0.0317
Median	2.25	2.68	
IQR	0.7-5.3	1.1-5.5	

^∗^Significant differences. SD: standard deviations; IQR: interquartile range.

**Table 2:** Evaluation of hematological and serum biochemical parameters.

Cases	Control		*pvalue*	Propolis		*pvalue*
Subjects' number	8			20		
	Initial	Final		Initial	Final	
Creatinine (mg/dL)	1.53 ± 0.76	1.5 ± 1.14	0.9545	1.57 ± 2.1	1.73 ± 1.83	0.9828
Total cholesterol (mg/dL)	174 ± 70	179 ± 55	0.9560	161 ± 53	137 ± 24	0.6862
Triglycerides (mg/dL)	170 ± 40	170 ± 85	0.9999	165 ± 123	143 ± 53	0.8719
Glycemia (mg/dL)	321 ± 162	197 ± 98	0.5231	210 ± 87	215 ± 135	0.9756
HbA1C (%)	10.3 ± 3.2	9.2 ± 2.9	0.8026	9.6 ± 2.7	9.3 ± 2.4	0.9350
Hematocrit (%)	37.5 ± 7.1	36 ± 5.9	0.8732	37.5 ± 5.3	34.5 ± 7.2	0.7422
Hemoglobin (g/dL)	12.3 ± 2.33	11.8 ± 2.1	0.8756	12.5 ± 1.86	11.3 ± 2.5	0.7059
White blood cells (mm^3^)	9543 ± 3703	8726 ± 4917	0.8963	8811 ± 3215	9286 ± 1774	0.8916

## Data Availability

Due to ethical concerns, supporting data cannot be made openly available and are restricted by the committee of the Maule Health Service according to Chilean Law No. 25584 in order to protect patient privacy. The data are available from Dra. Verónica Mujica as the treating doctor and Jessica Zuñiga-Hernandez as the corresponding author, for researchers who meet the criteria for access to this confidential data.

## Supplementary Materials

Supplementary Materials Figure S1: consolidate standard of reporting trial (CONSORT) flow chart with the participant recruitment and progress through a study. Table S1: foot care and dressing. List of dressings used for wound care by a specialist nurse in advanced healing. Both groups received the same type of protocol. The protocols were adjusted to the technical standards of the Ministry of Health, Minsal, Chile, and were ranked according to the degree of exudate and/or presence of infection. Table S2: arbitrary connective tissue (ACT) score. Based on Ishak17. Table S3: concomitant pathologies and chronicled pharmacology therapy. Figure S2: serum laboratory analysis of glycaemia (A), HbA1c (B), and usPCR (C). Table S4: determination of microorganisms present in the diabetic foot wound. Negative culture means biopsy culture does not show growth of bacteria or fungi.
