# Supplementary material for: Propolis as an Adjuvant in the Healing of Human Diabetic Foot Wounds Receiving Care in the Diagnostic and Treatment Centre from the Regional Hospital of Talca
Source: J Diabetes Res. 2019 Sep 12;2019:2507578. doi: 10.1155/2019/2507578 (PMC6757282; doi:10.1155/2019/2507578)
Supplement: Supplementary Materials — Figure S1: consolidate standard of reporting trial (CONSORT) flow chart with the participant recruitment and progress through a study. Table S1: foot care and dressing. List of dressings used for wound care by a specialist nurse in advanced healing. Both groups received the same type of protocol. The protocols were adjusted to the technical standards of the Ministry of Health, Minsal, Chile, and were ranked according to the degree of exudate and/or presence of infection. Table S2: arbitrary connective tissue (ACT) score. Based on Ishak17. Table S3: concomitant pathologies and chronicled pharmacology therapy. Figure S2: serum laboratory analysis of glycaemia (A), HbA1c (B), and usPCR (C). Table S4: determination of microorganisms present in the diabetic foot wound. Negative culture means biopsy culture does not show growth of bacteria or fungi. [file 2507578.f1.docx]

Figure S1

Analysed (n=20)

Analysed (n=8)

Assessed for eligibility (n=31)

Lost to follow-up (n=0) at the end study

Allocated to **propolis group** (n= 20)

Lost to follow-up (n=2) at the end study

- Respiratory complication(n=1)
- Heart surgery (n=1)

Allocated to **control group** (n=11)

**Allocation**

**Analysis**

**Follow-Up**

**Enrollment and Follow up**


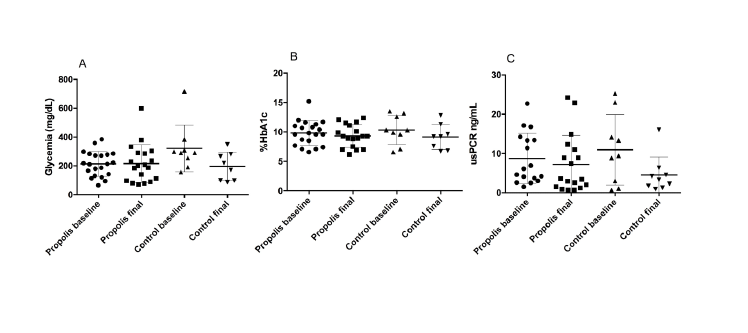
Figure S2

Table S1


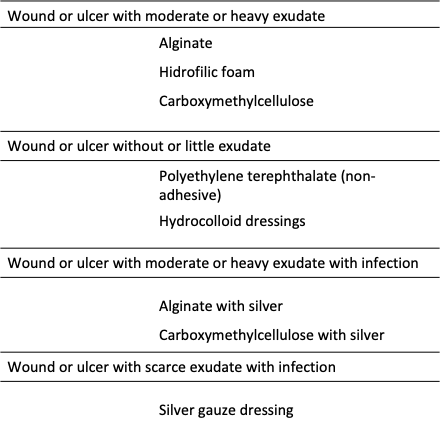


Table S2

| **Scale** | **Categorical Description** | **Percentage of deposit connective tissue** |
| --- | --- | --- |
| I | Vital (normal tissue) | Less than 5% |
| II | Fibrosis expansion (+/-) | Among 5 and 20% |
| III | Fibrosis expansion around vessel areas and connecting some of them | Among 20 and 40% |
| IV | Fibrosis expansion (marked bringing) | Among 40 and 80% |
| V | Cicatrized tissue | Over 80% or less tan 5% vital tissue |

Table S3

|  | Control | Propolis |
| --- | --- | --- |
| Concomitant pathologies |  |  |
| Obesity | 100% | 100% |
| Dyslipidemia | 62.5% (5 subjects) | 40%(8 subjects) |
| Hypertension | 75% (6 subjects) | 95% (19 subjects) |
| Coronary disease | 0% | 10% (2 subjects) |
| Chronicle kidney disease | 37.5 (3 subjects) | 30%(6 subjects) |
| Anemia | 0% | 5% (1 subject) |
| Asthma | 0% | 5% (1 subject) |
| Chronic pharmacological therapies |  |  |
| Insulin (NPH) | 100% | 100% |
| ASA | 25% (2 subjects) | 35% (7 subjects) |
| Statins (atorvastatin) | 62.5 (5 subjects) | 40% (8 subjects) |
| ARBs (losartan) | 75% (6 subjects) | 95%(19 subjects) |
| Others antihypertensive medications (amlodipine, carvedilol, hydrochlorothiazide, others) | 50% (4 subjects) | 50% (1º subjects) |
| Others therapies | 0% | 10% (2 subjects) |

NPH Neutral protamine Hagedorn insulin.

ASA acetyl salicylic acid

ARBs Angiotensin II receptor blockers

Table S4

|  | **Control** | **(%)** | **Propolis** | **(%)** |
| --- | --- | --- | --- | --- |
| **Types of bacteria present** | *S. aureus* | 33.4 | *S. aureus* | 30 |
|  | Streptococcus sp | 22.2 | *P. Aeruginosa* | 10 |
|  | *S. coagulasa* negative | 11.1 | Proteus sp | 5 |
|  | *E. Coli / K. pneumoniae* | 11.1 | Klebsiella sp | 5 |
|  | Negative culture | 22.2 | *S. Coagulasa* negative | 5 |
|  |  |  | Streptococcus sp | 5 |
|  |  |  | Negative culture | 40 |
| **Types of fungi present** | Negative culture |  | Negative culture |  |
